# Subregional hypothalamic volumes associate with childhood conduct problems in healthy adults

**DOI:** 10.1007/s11682-026-01154-6

**Published:** 2026-05-02

**Authors:** Assunta Pelagi, Chiara Camastra, Andrea Quattrone, Alessia Sarica

**Affiliations:** 1 Department of Medical and Surgical Sciences, Magna Graecia University, Catanzaro, 88100 Italy; 2 Neuroscience Research Center, Magna Graecia University, Catanzaro, 88100 Italy; 3 Institute of Neurology, Department of Medical and Surgical Sciences, Magna Graecia University, Catanzaro, 88100 Italy

**Keywords:** Hypothalamus subunits, Childhood conduct problems, Conduct disorder, Logistic regression, MRI

## Abstract

Childhood conduct problems have been linked to alterations in frontolimbic circuits, yet the contribution of hypothalamic subregions remains largely unexplored. This study examined whether hypothalamic morphometry relates to conduct problems in 600 healthy young adults from the Human Connectome Project (206 with and 394 without conduct problems). Volumes of twelve hypothalamic subregions were extracted from structural MRI using the automated FreeSurfer segmentation. Group differences were assessed with linear models, and multivariable associations were tested with logistic regression adjusted for age, sex, and intracranial volume. Influential observations were screened using Cook’s distance, and predictor importance was further evaluated through logistic LASSO regression with tenfold cross-validation. Individuals with a history of conduct problems showed larger volumes in anterior and tubular hypothalamic subregions—specifically the left anterior-inferior, left tubular-inferior, and right tubular-superior—while an initial inverse association in the left posterior subregion was no longer significant after excluding influential cases. These findings identify distinct hypothalamic subregions as structural correlates of early behavioral dysregulation and suggest that hypothalamic morphometry may reflect neurodevelopmental adaptations associated with childhood conduct problems.

**Supplementary Information:**

The online version contains supplementary material available at 10.1007/s11682-026-01154-6.

## Introduction

Childhood conduct problems (CC), including Conduct Disorder (CD) and Oppositional Defiant Disorder (ODD), represent a persistent pattern of aggressive, defiant, and antisocial behaviors that violate social norms and the rights of others. These behaviors encompass physical aggression, deceitfulness, property destruction, and rule violations. When persistent and severe, they constitute the clinical diagnosis of Conduct Disorder, a prevalent childhood psychiatric condition associated with substantial long-term functional impairments (Fairchild et al., 2019; Prinstein et al., 2019). ODD, which often precedes CD, typically emerges before age 8 and rarely after age 12, while CD is usually categorized by age of onset: childhood onset (before age 10) and adolescent onset (after age 10) (Behavior or Conduct Problems in Children | Children’s Mental Health | CDC, n.d.; Ontario Psychological Association - Conduct Disorder: Understanding the Spectrum of Behaviour in Children, n.d.). Importantly, even subclinical conduct problems, those not meeting diagnostic criteria, have been linked to an increased risk for later psychopathology, including antisocial personality disorder, substance abuse, and mood disturbances (Fisher et al., 2024). This suggests that early behavioral dysregulation may leave enduring neurodevelopmental traces detectable in adulthood.

From a neurobiological perspective, theories of conduct problems emphasize disruptions in brain circuits underlying emotion regulation, impulse control, and social cognition. Neuroimaging studies have consistently reported structural and functional alterations in frontolimbic regions, including reduced volume or atypical activation in the amygdala, orbitofrontal cortex, anterior cingulate cortex, and hippocampus (Gao et al., 2024; Gouveia et al., 2019). However, a growing body of evidence indicates that subcortical and diencephalic structures, particularly the hypothalamus, may also play a critical yet underexplored role in behavioral regulation and aggression control (Gouveia et al. 2023a, b; Wang et al. 2025; Yao et al. 2024).

The hypothalamus is a central hub regulating autonomic, endocrine, and behavioral responses to salient environmental cues. Through its coordination of the hypothalamic–pituitary–adrenal (HPA) axis, it orchestrates physiological stress responses, emotional reactivity, and social threat detection (Fairchild et al. 2018; Gouveia et al. 2019, 2023a, b; Saper and Lowell 2014; Yao et al. 2024). Experimental evidence indicates that specific hypothalamic nuclei, such as the ventromedial and posterior regions, are crucial for aggressive and defensive behaviors in animal models (Mei et al., 2023; Toth et al., 2010; Yang et al., 2023). In humans, stimulation of posterior hypothalamic areas has been shown to modulate pathological aggression, while altered hypothalamic connectivity has been linked to impulsivity and reactive aggression in youths (Benedetti-Isaac et al., 2023; Yao et al., 2025).

Despite this well-established functional relevance, the structural anatomy of the hypothalamus remains comparatively underexplored in human neuroimaging studies of conduct-related behavior. Most previous work has examined cortical and limbic correlates in clinical or forensic samples, leaving open the question of whether structural differences in hypothalamic subregions may also reflect developmental traces of early behavioral dysregulation. An additional challenge stems from the hypothalamus’s complexity. It is a small but heterogeneous structure composed of nuclei with distinct physiological and behavioral roles (Lechan & Toni, 2016). Recent advances in automated segmentation now allow for its parcellation into anatomically and functionally defined subregions (Billot et al., 2020), enabling more precise assessment of its relationship with behavior. Evidence suggests that anterior and tubular regions are involved in emotional and social threat processing, whereas posterior regions contribute more directly to motor aspects of aggression and arousal regulation (Goel et al., 2025; Saper & Lowell, 2014). Demographic moderators may further modulate these associations. Conduct problems are consistently more prevalent among males (Álvarez-Voces & Romero, 2025; Brooks Holliday et al., 2017; Lindner et al., 2016), and sex differences in hypothalamic maturation have been linked to variations in emotional and stress regulation (Xu et al., 2025). Moreover, age-related changes in hypothalamic morphology across young adulthood may reflect persistence or compensatory adaptation following childhood-onset difficulties (Essex et al., 2011; Xu et al., 2025).

Given these considerations, the present study investigated hypothalamic morphometry at the subregional level in a large cohort of young adults from the Human Connectome Project. Participants were classified according to a retrospective history of childhood conduct problems. We examined whether specific hypothalamic subregions were associated with conduct-problem status while statistically controlling for age, sex, and intracranial volume. To ensure robust inference and minimize overfitting, both standard and regularized logistic regression models were employed. By focusing on subregional hypothalamic morphology within a healthy population, this study seeks to clarify whether individual differences in hypothalamic structure are linked to early externalizing tendencies, offering new insight into their possible neurodevelopmental underpinnings.

## Methods

### Participants

Data for this study were obtained from the publicly available and restricted S1200 release of the Human Connectome Project – Young Adult (HCP-YA) dataset (Van Essen et al., 2013). The HCP, a collaborative effort led by Washington University, the University of Minnesota, and the University of Oxford, aims to map human brain networks and their behavioral correlates comprehensively. It includes a large cohort of healthy young adults assessed with advanced multimodal imaging and behavioral measures, integrating structural and diffusion MRI with extensive phenotypic and cognitive data (Van Essen et al., 2013).

Data files in.csv format were downloaded on December 11th, 2023. The open-source analytics platform KNIME version 4.6.1 (Sarica et al., 2014) was used to filter and merge relevant HCP tables and to remove cases with missing data.

Following prior evidence indicating that brain morphology may vary across ethnic groups, potentially influencing neuroimaging analyses (Atilano-Barbosa & Barrios, 2023; Ioannou et al., 2022), only participants self-identified as White (the largest subgroup in the HCP cohort) were included to ensure sample homogeneity. The final sample comprised 630 individuals (290 males and 340 females).

Participants were classified into two groups based on a retrospective history of childhood conduct problems, as assessed using the Semi-Structured Assessment for the Genetics of Alcoholism (SSAGA; Bucholz et al., 1994). Individuals reporting at least one childhood conduct problem before age 15 were coded as CC, and all others as HC (healthy control). This liberal definition was adopted to capture subclinical conduct tendencies within a non-clinical population, in line with previous population-based studies that similarly operationalized subclinical conduct traits using low symptom thresholds (Abdolalizadeh et al., 2023).

To evaluate the robustness of this definition, a sensitivity analysis was conducted using a more stringent threshold (≥ 2 symptoms), as described below.

In addition to behavioral history, general cognitive and emotional-behavioral measures available from the HCP dataset were considered to characterize the sample. These included selected scores from the NIH Toolbox Cognitive Battery (e.g., fluid intelligence, working memory) and the NIH Emotion Battery (e.g., anger affect, fear affect, sadness), which provide standardized indices of cognitive performance and emotional traits relevant to behavioral regulation.

### MRI acquisition and processing

Structural MRI data were obtained from the Human Connectome Project – Young Adult (HCP-YA) dataset. Images were acquired on a 3 T Siemens Skyra scanner using the HCP standardized acquisition protocol, providing high-resolution T1-weighted scans with 0.7 mm isotropic voxels (Van Essen et al., 2013). This protocol ensures superior anatomical detail compared to conventional clinical MRI acquisitions.

From the HCP’s Amazon S3 repository, we downloaded the extended structural preprocessed directories, which include intermediate outputs generated by the standard recon-all pipeline of FreeSurfer v5.3.0 (Fischl, 2012; Glasser et al., 2013). This pipeline performs tissue classification (gray matter, white matter, cerebrospinal fluid) and cortical/subcortical segmentation from T1-weighted images.

Within these directories, hypothalamic subregion segmentation was performed using the automated tool implemented in FreeSurfer v7.2, which delineates twelve subregions: left and right anterior-inferior, anterior-superior, posterior, tubular-inferior, and tubular-superior, as well as total left and right hypothalamic volumes (Billot et al., 2020).

All volumetric measures are reported in cubic millimeters (mm³). Each segmentation was visually inspected for quality control, and participants showing clear segmentation errors were excluded from further analyses.

### Statistical analysis

All statistical analyses were conducted in R (version 4.2.2) and Jamovi (version 2.3). A complete-case dataset was used after quality control, excluding participants with missing or erroneous hypothalamic segmentations (e.g., ROI volumes equal to zero). Differences in cognitive and emotional measurements were evaluated using ANCOVA with sex, age, and education as covariates. Partial eta-squared (η²ₚ) was reported as a measure of effect size. Assumptions were assessed using Levene’s test and interaction terms; where violations were detected, heteroskedasticity-robust standard errors (HC3) were applied. Family-wise error was controlled using Bonferroni correction.

Also group differences in hypothalamic subregional volumes were evaluated with ANCOVA with sex, age, and intracranial volume (ICV) as covariates. Partial eta-squared (η²ₚ) was reported as a measure of effect size. To control for multiple comparisons across the 12 subregions, Bonferroni correction was applied to maintain the family-wise error rate.

We then fitted a multivariable binomial logistic regression (outcome: CC vs. HC), including all subregional volumes and covariates (age, sex, ICV). To mitigate multicollinearity, highly correlated ROIs (|r| > 0.80) were excluded from multivariable analyses (Dormann et al., 2013). Continuous covariates (age and intracranial volume, ICV) were z-standardized before modeling. Education did not differ between groups and was not included as a covariate in the primary ROI models or logistic analyses.

Multicollinearity was further assessed using variance inflation factors (VIF). Model discrimination was quantified by the area under the receiver operating characteristic curve (AUC). Calibration assessment indicated acceptable agreement between predicted probabilities and observed outcomes.

Influential observations were identified using Cook’s distance (threshold = 4/n; Cook, 1977), and the model was re-estimated after excluding them. Sensitivity analyses were additionally performed by re-estimating all models using a stricter definition of childhood conduct problems (≥ 2 symptoms).

Because all hypothalamic subregions were modeled jointly within a single pre-specified anatomical hypothesis space, p-values for ROI terms in the multivariable model are reported as nominal, and the logistic analysis is presented as a secondary, hypothesis-driven complement to the primary group-mean comparisons. However, to assess the robustness of ROI-specific effects, false discovery rate (FDR) correction was additionally explored in supplementary analyses.

Finally, to rank the relative contribution of each ROI while minimizing overfitting, a logistic LASSO regression with 10-fold cross-validation was performed (Friedman et al., 2010), keeping covariates unpenalized. Effects were expressed as odds ratios per one standard deviation of each retained predictor.

## Results

### Cohort characteristics

The initial dataset comprised 630 individuals, but after quality control of hypothalamic segmentations, 30 participants showing missing or null ROI volumes were excluded. The final complete-case cohort comprised 600 individuals (394 HC and 206 CC; 290 males, 340 females) with a mean age of 29 years (range: 22–36). In the sensitivity analysis using a stricter threshold (≥ 2 symptoms), the sample size remained unchanged (*N* = 600), but the CC group was reduced to 51 participants (HC = 549). Global cognitive performance was within the normal range (Mini-Mental State Examination [MMSE] mean = 29, range: 23–30). To further characterize the sample, selected cognitive and emotional-behavioral measures from the HCP dataset were examined, including, for example, fluid intelligence, working memory (NIH Toolbox Cognitive Battery), and affective traits such as anger, fear, and sadness (NIH Emotion Battery). The CC group showed higher scores on anger facets (anger affect, hostility, aggression) and perceived hostility; these differences are consistent with the conduct-problem phenotype. Small cognitive differences were also observed (e.g., vocabulary and composite scores), with small effect sizes. Although these cohort characteristics are reported for transparency and are not the primary focus of the morphometric analyses, the findings indicate that the groups differed primarily in anger-related traits, consistent with the conduct-problem phenotype, while general cognitive functioning was largely comparable. Table 1 summarizes the demographic and cognitive characteristics of the HCP cohort by group (HC vs. CC).

**Table 1:** Descriptive characteristics of the HCP young adult cohort by group (HC vs. CC)

	Group		Mean (or frequencies)	Median	SD	Min	Max
Male/Female	HC	137/257		-	-	-	-
	CC	138/68		-	-	-	-
Age	HC	29.2		29	3.55	22	36
	CC	29.1		29	3.36	22	36
Education (in years)	HC	15.2		16	1.63	11	17
	CC	15.1		16	1.69	11	17
MMSE	HC	29.1		29	0.93	26	30
	CC	29.0		29	1.03	23	30
ICV	HC	1.58 × 10^6^		1.57 × 10^6^	1.71 × 10⁵	1.07 × 10^6^	2.04 × 10^6^
	CC	1.65 × 10^6^		1.67 × 10^6^	1.69 × 10⁵	1.18 × 10^6^	2.14 × 10^6^
left anterior-inferior	HC	13.4		13.3	4.39	1.58	28.9
	CC	14.3		14.6	4.63	2.97	26.5
left anterior-superior	HC	21.7		21.5	4.03	9.15	34.2
	CC	22.5		22.6	4.16	8.47	35.4
left posterior	HC	107.1		107.5	13.88	59.82	152.9
	CC	110.3		109.0	14.36	67.47	155.7
left tubular-inferior	HC	131.7		130.4	18.28	70.94	180.7
	CC	139.9		140.8	18.18	83.14	179.1
left tubular-superior	HC	110.7		110.2	14.40	71.59	158.1
	CC	116.8		116.2	15.98	38.57	154.7
right anterior-inferior	HC	12.9		12.9	4.85	1.18	27.4
	CC	13.7		13.7	4.41	1.34	23.8
right anterior-superior	HC	22.0		21.6	4.72	7.73	39.6
	CC	22.5		22.1	4.82	11.45	39.5
right posterior	HC	115.9		116.0	16.28	64.37	171.6
	CC	118.8		117.8	16.02	77.71	161.5
right tubular-inferior	HC	125.3		123.1	18.20	71.12	178.2
	CC	132.7		135.0	18.63	70.70	184.5
right tubular-superior	HC	114.3		114.3	13.89	79.45	163.7
	CC	119.3		120.0	15.28	78.39	162.9
whole left	HC	384.6		381.3	42.23	277.53	497.0
	CC	403.8		406.4	43.76	271.16	499.2
whole right	HC	390.4		388.7	41.48	287.09	512.3
	CC	407.4		411.2	42.57	303.76	509.5

*CC* Childhood conduct problems, *HC* Healthy Control, *MMSE* Mini-Mental State Examination, *ICV *Intracranial Volume, *SD* Standard Deviation, *Min* Minimum, *Max *Maximum

### Group differences in hypothalamic volumes

**Fig. 1:**
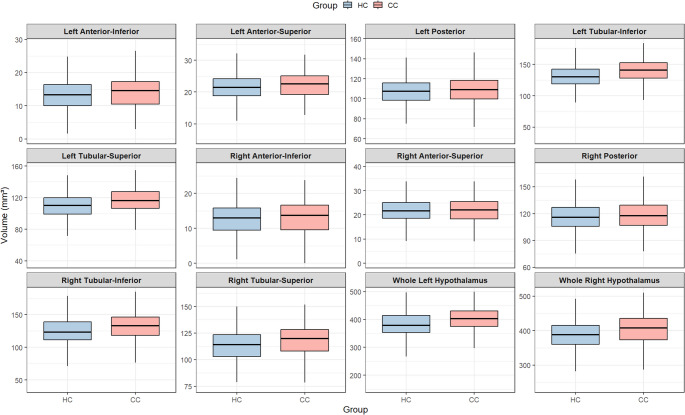
Boxplot distribution of hypothalamic subregion volumes (mm³) in participants with (CC) and without (HC) childhood conduct problems. The CC group shows larger volumes in several subregions, notably in the tubular areas and the whole hypothalamus

Group differences in hypothalamic subregional volumes were first examined visually using box plots (Fig. 1).

Inferential statistics were obtained from ANCOVA with sex, age, and ICV as covariates. A Bonferroni correction was applied to control for multiple comparisons across the 12 subregions. Results are summarized in Table 2. Effect sizes were in the small-to-moderate range, with the largest effects observed for the whole left hypothalamus (η²ₚ = 0.072) and left tubular-inferior region (η²ₚ = 0.063), followed by whole right (η²ₚ = 0.053) and bilateral tubular subregions (η²ₚ ≈ 0.04–0.05). The CC group exhibited larger volumes across several subregions, particularly in tubular and whole hypothalamic regions. After correction, significant differences were observed for the left posterior (*p*=.022), left tubular-inferior, left tubular-superior, right tubular-inferior, right tubular-superior, and whole left and right hypothalamus (all *p* <.001, Bonferroni-adjusted). Differences in the left anterior-inferior and right posterior subregions did not survive correction. No statistically significant group effects were detected for the left anterior-superior, right anterior-inferior, or right anterior-superior regions.

**Table 2:** Group differences in hypothalamic region-of-interest (ROI) volumes (CC vs. HC)

Subregion	Mean HC	Mean CC	partial η²	*p*-value	p_Bonf
left anterior-inferior	13.55	14.18	0.002	0.011	0.137
left anterior-superior	21.96	22.10	0.011	0.688	1.000
left posterior	109.02	107.65	0.016	0.002	**0.022**
left tubular-inferior	134.45	136.14	0.049	< 0.001	**< 0.001**
left tubular-superior	112.72	113.88	0.050	< 0.001	**< 0.001**
right anterior-inferior	13.16	13.32	0.006	0.058	0.699
right anterior-superior	22.12	22.30	0.011	0.681	1.000
right posterior	117.41	116.44	0.009	0.020	0.239
right tubular-inferior	127.93	129.07	0.049	< 0.001	**< 0.001**
right tubular-superior	115.24	117.96	0.039	< 0.001	**< 0.001**
whole left	391.70	393.45	0.072	< 0.001	**< 0.001**
whole right	395.86	399.08	0.053	< 0.001	**< 0.001**

Significant p-values (p <.05) are highlighted in bold

### Logistic and LASSO regression models

A binomial logistic regression was performed to examine whether hypothalamic subregional volumes predicted group membership (CC vs. HC). All subregional volumes were entered as predictors, with age (z-scored), sex, and intracranial volume (ICV, z-scored) included as covariates.

Due to high collinearity among variables (|r| > 0.80), the whole left and whole right hypothalamic volumes were excluded from the multivariable model. Influential observations were identified using Cook’s distance (threshold = 4/n; Cook, 1977), resulting in the removal of 19 cases (18 CC and 1 HC). The model was then re-estimated after excluding these cases. Variance inflation factors indicated no evidence of problematic multicollinearity (all VIFs < 3.2; Table S3, Supplementary Material). FDR correction was explored in supplementary analyses (Table S2).

As illustrated in Figure S1 (Supplementary Material), comparison of the receiver operating characteristic (ROC) curves with and without influential observations confirmed improved discrimination for the final model (AUC = 0.75, compared with 0.71 before exclusion). Based on Youden’s index, the optimal classification threshold was 0.392, yielding accuracy = 72.1% (95% CI 68.4–75.7), sensitivity = 64.5%, specificity = 75.9%, and balanced accuracy = 70.2%.

Table 3 reports the odds ratios (OR), 95% confidence intervals (CI), and p-values for the final cleaned model. Because all hypothalamic subregions were modeled jointly within a single pre-specified anatomical hypothesis space, p-values for ROI terms are reported as nominal, and the logistic analysis is presented as a secondary, hypothesis-driven complement to the primary group-mean comparisons.

**Table 3:** Multivariable logistic regression (“cleaned” dataset after Cook’s distance) predicting CC (vs. HC)

Variable	Odds Ratio	95% CI (Lower–Upper)	*p*-value
Intercept	0.011	0.001–0.170	0.001
Left Anterior-Inferior	1.073	1.016–1.133	**0.012**
Left Anterior-Superior	0.964	0.900–1.031	0.290
Left Posterior	0.979	0.957–1.002	0.070
Left Tubular-Inferior	1.025	1.006–1.045	**0.010**
Left Tubular-Superior	0.993	0.972–1.015	0.535
Right Anterior-Inferior	1.009	0.952–1.070	0.773
Right Anterior-Superior	0.988	0.932–1.047	0.686
Right Posterior	0.997	0.980–1.015	0.769
Right Tubular-Inferior	0.992	0.973–1.011	0.393
Right Tubular-Superior	1.034	1.014–1.056	**< 0.001**
Sex (Male)	4.866	2.760–8.727	**< 0.001**
Age	1.072	1.011–1.137	**0.021**
Intracranial Volume	0.907	0.676–1.217	0.515

Significant p-values (p <.05) are highlighted in bold

The logistic regression model identified three hypothalamic subregions significantly associated with childhood conduct problems. Larger volumes in the right tubular-superior (OR = 1.034, 95% CI [1.014–1.056], *p* <.001), left tubular-inferior (OR = 1.025, 95% CI [1.006–1.045], *p* =.010), and left anterior-inferior (OR = 1.073, 95% CI [1.016–1.133], *p* =.012) were linked to higher odds of belonging to the CC group. The left posterior subregion showed a small inverse trend that did not reach significance after excluding influential observations (OR = 0.979, 95% CI [0.957–1.002], *p* =.070). Among covariates, male sex (OR = 4.866, 95% CI [2.760–8.727], *p* <.001) and age (OR = 1.072, 95% CI [1.011–1.137], *p* =.021) were significant predictors, whereas intracranial volume (ICV) was not (OR = 0.907, 95% CI [0.676–1.217], *p* =.515). No other hypothalamic subregions significantly contributed to group classification.

**Fig. 2:**
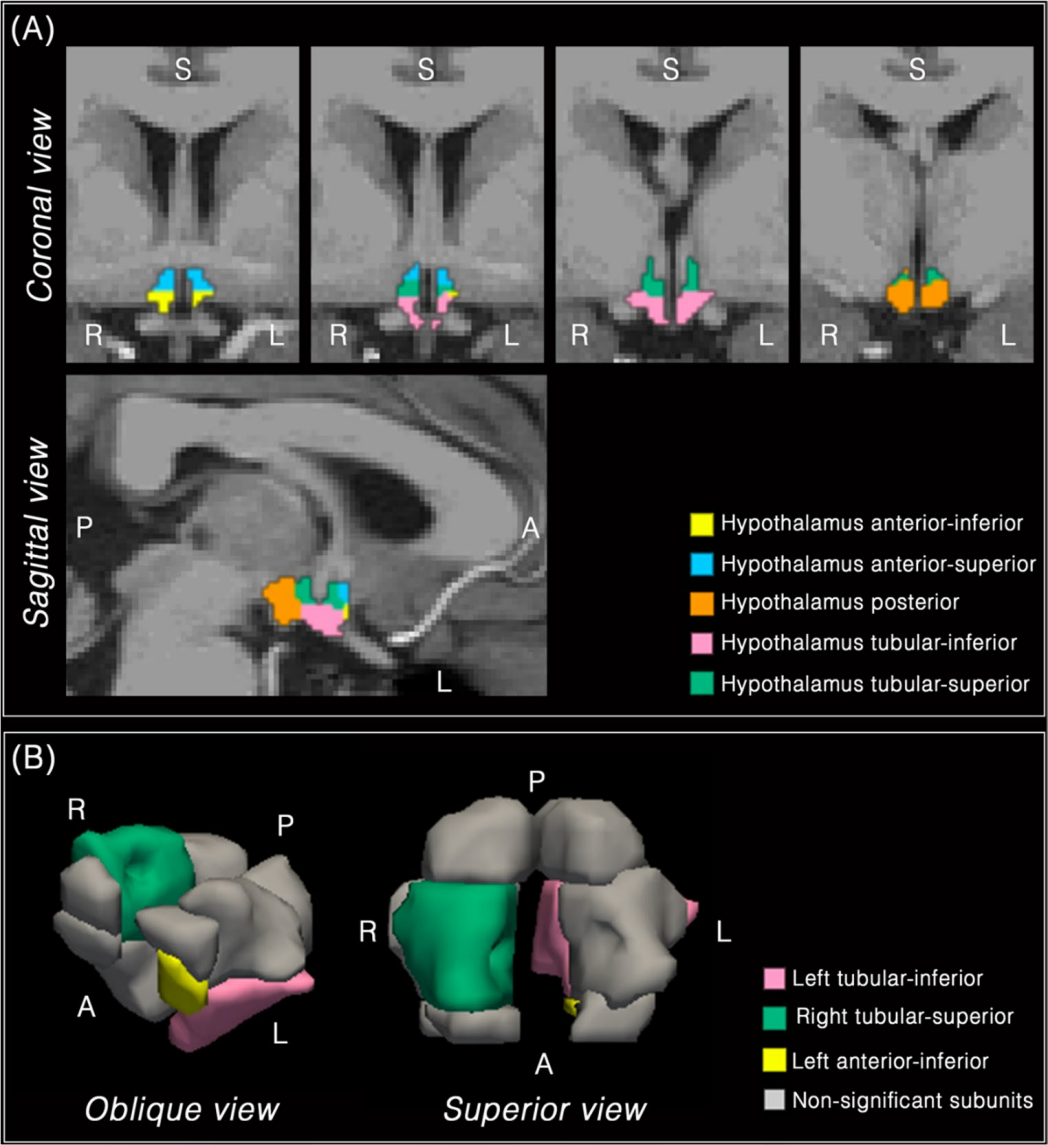
(**A**) Representative segmentation of hypothalamic subregions in a healthy control subject, displayed for anatomical reference in coronal and sagittal views. (**B**) Logistic regression results showing hypothalamic subregions significantly associated with childhood conduct problems (CC). Larger volumes in the right tubular-superior (green), left tubular-inferior (pink), and left anterior-inferior (yellow) subregions were linked to higher odds of belonging to the CC group. Non-significant hypothalamic subunits are shown in light gray for context

Figure 2 provides a visual summary of the logistic regression results. Panel A shows the segmentation of hypothalamic subregions in a representative healthy control, serving as an anatomical reference. Panel B displays the subregions that emerged as significant predictors of childhood conduct-problem (CC) status after multivariable adjustment, highlighting their spatial distribution within the anterior and tubular portions of the hypothalamus. Non-significant subunits are shown in light gray for anatomical context.

Together, these results indicate that volumetric variation in anterior and tubular hypothalamic subregions contributes meaningfully to distinguishing individuals with a history of childhood conduct problems from controls. To further evaluate the relative importance of these predictors while reducing the risk of model overfitting, we next applied a logistic LASSO regression with tenfold cross-validation (covariates unpenalized; results reported at λ = lambda.min). The model was used for feature selection and ranking rather than formal statistical inference.

Effect sizes were expressed as odds ratios (OR) per one standard deviation (Figure S2, Supplementary Material). The most influential predictors were male sex (≈ 1.99), age (≈ 1.16), right tubular-superior (≈ 1.16), left posterior (≈ 0.92; inverse), left anterior-inferior (≈ 1.08), and left tubular-inferior (≈ 1.02). This pattern closely mirrored the results from the logistic regression model, supporting the robustness of the identified associations.

Sensitivity analyses using a more stringent definition of conduct problems (≥ 2 symptoms; Table S1, Supplementary Material) yielded directionally consistent results for the main effects, although statistical significance was reduced. In ANCOVA models, significant group differences after Bonferroni correction were retained for the whole left hypothalamus and the left tubular-inferior region.

In logistic regression models, overall discrimination remained comparable (AUC ≈ 0.75), although individual coefficients were less stable and fewer regions reached statistical significance. This attenuation is likely attributable to the substantial reduction in the CC group (*n* = 51), resulting in decreased statistical power.

## Discussion

This study investigated the association between hypothalamic subregional morphology and retrospectively reported childhood conduct problems in a large cohort of healthy young adults. Participants with a history of such problems exhibited structural differences in specific hypothalamic subregions. In multivariable models controlling for age, sex, and intracranial volume, larger volumes in the left anterior-inferior, left tubular-inferior, and right tubular-superior subregions were associated with higher odds of belonging to the conduct-problem group. An initially observed inverse association for the left posterior subregion attenuated and lost significance after removal of influential observations.

Sensitivity analyses using a stricter definition of conduct problems (≥ 2 symptoms) indicated that the main findings were partially robust, particularly for the left hypothalamus and tubular subregions, although reduced statistical power likely limited the stability of multivariable estimates. Importantly, the consistency of the direction of effects across different thresholds supports the robustness of the observed associations and suggests that the findings are not driven by the specific operational definition of conduct problems.

The hypothalamus plays a central role in stress responsivity, emotional regulation, social behavior, and aggression, domains frequently affected in individuals with conduct problems and related externalizing traits (Bell et al., 2024; Fairchild et al., 2019; Gouveia et al., 2019; Yao et al., 2024). The observed pattern of larger volumes in anterior and tubular subregions aligns with emerging evidence that hypothalamic development is sensitive to early behavioral and emotional difficulties. For instance, Wang et al. (2025) reported accelerated hypothalamic growth in children with elevated behavioral problems, suggesting that such enlargement may reflect compensatory or maturational adaptations following early adversity. These subregions are anatomically and functionally involved in integrating social threat cues, coordinating neuroendocrine responses, and supporting affiliative behaviors (Saper & Lowell, 2014), offering a plausible framework to interpret the present findings. In this context, integrating structural measures with functional and connectivity-based information may provide a more comprehensive characterization of brain–behavior relationships, as illustrated by recent multimodal neuroimaging approaches combining structural and functional connectomes (Ghosh, Raj et al., 2023).

However, the present cross-sectional design does not allow causal or developmental inferences. The observed volumetric differences may reflect multiple, not mutually exclusive mechanisms, including delayed maturation, stress-related neuroendocrine adaptation, or compensatory developmental processes.

The posterior hypothalamus has been consistently implicated in the modulation of aggressive behavior in both lesion and deep-brain stimulation studies(Gouveia et al. 2019, 2023a, b). In the present study, the direction of the effect observed for the left posterior subregion was consistent with this literature, but it fell below conventional significance after exclusion of high-leverage cases, most of which belonged to the CC group. In the LASSO analysis, the same inverse directionality was retained, supporting the view that posterior hypothalamic morphology may reflect individual variability in aggression-related control circuits. Previous work has also highlighted structural variability in subcortical brain regions within the HCP cohort, supporting the relevance of morphometric approaches in capturing individual differences (Camastra et al., 2025).

Beyond brain structure, cohort profiling showed the expected group differences in anger-related traits (e.g., hostility, aggression), which are conceptually aligned with conduct-problem phenotypes and with broader dimensions of psychological functioning and well-being (Pelagi et al., 2025).

Small differences between groups were also observed for some cognitive indices (e.g., crystallized intelligence), with a small effect size; these differences were not the focus of the study and are unlikely to fully account for the hypothalamic findings. Together, these results support the interpretation that behaviorally relevant hypothalamic variation co-occurs with conduct-related traits rather than reflecting broad cognitive impairment.

Demographic covariates also contributed meaningfully to group classification. Older age showed a modest positive association with CC status, which may reflect either cumulative neurodevelopmental effects of early behavioral difficulties or age-related differences in retrospective recall (Groß & Bayen, 2021). Consistent with epidemiological findings, male sex was strongly associated with conduct-problem status (Yao et al., 2024). This sex-related difference may arise from both biological and social mechanisms, including sex-differentiated hypothalamic maturation and neuroendocrine modulation (e.g., HPA axis), with downstream effects on stress reactivity, aggression, and emotional regulation (Fairchild et al., 2018, 2019; Goel et al., 2014; Uhart et al., 2006).

Most previous neuroimaging research on conduct problems has focused on larger limbic and frontal structures, such as the amygdala and orbitofrontal cortex, typically reporting reduced volumes or delayed maturation (Abdolalizadeh et al., 2023; Gao et al., 2024). The present findings extend this literature by highlighting hypothalamic morphometry as an underexplored yet behaviorally relevant target in individuals with conduct-related traits. For example, Bell et al. (2024) reported reduced hypothalamic volumes in violent forensic patients with psychotic comorbidities, whereas the pattern observed in our community-based, non-clinical cohort may reflect a distinct neurodevelopmental trajectory, potentially characterized by adaptive or compensatory structural variations rather than overt neuropathology.

Several limitations should be acknowledged. First, the retrospective classification of childhood conduct problems is inherently vulnerable to recall bias and cannot substitute for a formal clinical assessment. Second, although the Human Connectome Project provides exceptionally high-quality imaging and detailed phenotyping, its focus on high-functioning participants without major psychiatric or neurological disorders may limit the generalizability of our findings to clinical or forensic populations. Third, the sample was restricted to White participants to enhance homogeneity, which improves internal validity but limits generalizability. Replication in independent and ethnically diverse samples will be essential to confirm the generalizability of these findings. Fourth, the cross-sectional design precludes causal inference; it remains unclear whether hypothalamic variations precede, accompany, or follow conduct-related difficulties. Finally, the automated segmentation of hypothalamic subregions, although validated and widely adopted, remains constrained by the spatial resolution of MRI and the precision of current parcellation algorithms. In this regard, recent work has highlighted the potential of multimodal structural MRI approaches for improving the characterization of neuroimaging biomarkers, including applications to joint classification and severity estimation in clinical populations (Lin et al., 2023).

Future longitudinal studies integrating neuroendocrine, behavioral, and developmental measures, as well as graph-based and multimodal modeling approaches, are needed to determine whether hypothalamic morphometry represents a marker of resilience, compensation, or vulnerability to persistent dysregulation (Ghosh et al. 2023a, b).

## Conclusions

In summary, specific hypothalamic subregions, particularly the left anterior-inferior, left tubular-inferior, and right tubular-superior, were larger in adults who retrospectively reported childhood conduct problems. These volumetric enlargements were associated with higher odds of conduct-problem status after adjusting for age, sex, and intracranial volume. Together with convergent LASSO rankings, these findings extend the neurodevelopmental framework of externalizing behavior beyond corticolimbic circuits and highlight the hypothalamus as a plausible diencephalic hub linking stress responsivity, social behavior, and aggression control.

Importantly, the observed pattern in this non-clinical cohort suggests that hypothalamic variation may not simply reflect pathology, but could be consistent with compensatory or adaptive processes; however, this interpretation remains speculative and requires longitudinal confirmation. This interpretation resonates with recent developmental evidence pointing to accelerated hypothalamic maturation following early adversity. Integrating such morphometric data with functional imaging, endocrine profiling, and longitudinal behavioral assessments will be essential to disentangle whether these differences confer resilience or mark vulnerability to persistent dysregulation.

Beyond its immediate implications, this work underscores the value of examining subcortical and diencephalic structures within the broader landscape of social and emotional neurodevelopment. By combining high-resolution morphometry with interpretable machine learning, future research can move toward more individualized models of behavioral risk, linking structure, function, and hormonal dynamics to better understand the neural architecture underlying externalizing tendencies.

## Supplementary Information

Below is the link to the electronic supplementary material.

Supplementary Material 1 (DOCX 140 KB)

## Data Availability

Data used in this study are available from the WU-Minn Human Connectome Project (Young Adult S1200 release) upon registration and agreement to the data-use terms.

## Code Availability

All analysis code is available from the corresponding author on reasonable request.
