# Supplementary material for: Subregional hypothalamic volumes associate with childhood conduct problems in healthy adults
Source: Brain Imaging Behav. 2026 May 2;20(3):85. doi: 10.1007/s11682-026-01154-6 (PMC13135012; doi:10.1007/s11682-026-01154-6)
Supplement: Supplementary file 1 — Supplementary Material 1 (DOCX 140 KB) [file 11682_2026_1154_MOESM1_ESM.docx]

Subregional Hypothalamic Volumes Associate with Childhood Conduct Problems in Healthy Adults

SUPPLEMENTARY MATERIAL

**Table S1.** Sensitivity analysis using a stricter definition of conduct problems (≥2 symptoms).

**Panel A.** ANCOVA results.

| **ROI** | **Adj_Mean_HC** | **Adj_Mean_CC** | **p_group** | **p_Bonf** | **partial η²** |
| --- | --- | --- | --- | --- | --- |
| **Left tubular-inferior** | 135.030 | 135.240 | 0.002 | **0.018** | 0.017 |
| **Left posterior** | 108.520 | 108.690 | 0.007 | 0.089 | 0.012 |
| **Left anterior-superior** | 21.960 | 22.560 | 0.024 | 0.285 | 0.009 |
| **Left anterior-inferior** | 13.750 | 14.020 | 0.309 | 1.000 | 0.002 |
| **Left tubular-superior** | 113.440 | 110.000 | 0.342 | 1.000 | 0.002 |
| **Right anterior-inferior** | 13.190 | 13.440 | 0.204 | 1.000 | 0.003 |
| **Right anterior-superior** | 22.140 | 22.640 | 0.239 | 1.000 | 0.002 |
| **Right posterior** | 117.330 | 114.380 | 0.644 | 1.000 | 0.000 |
| **Right tubular-inferior** | 128.600 | 125.650 | 0.228 | 1.000 | 0.004 |
| **Right tubular-superior** | 116.250 | 115.770 | 0.181 | 1.000 | 0.003 |
| **Whole left** | 392.700 | 390.500 | 0.001 | **0.018** | 0.017 |
| **Whole right** | 397.510 | 391.890 | 0.335 | 1.000 | 0.005 |

**Panel B.** Logistic regression.

| **Variable** | **OR** | **95% CI** | **p-value** |
| --- | --- | --- | --- |
| **Left anterior-inferior** | 1.033 | 0.949–1.123 | 0.446 |
| **Left anterior-superior** | 1.053 | 0.948–1.166 | 0.326 |
| **Left posterior** | 1.015 | 0.981–1.050 | 0.387 |
| **Left tubular-inferior** | 1.026 | 0.997–1.057 | 0.086 |
| **Left tubular-superior** | 0.954 | 0.922–0.986 | **0.006** |
| **Right anterior-inferior** | 0.992 | 0.908–1.086 | 0.853 |
| **Right anterior-superior** | 1.000 | 0.913–1.094 | 0.994 |
| **Right posterior** | 0.976 | 0.950–1.002 | 0.073 |
| **Right tubular-inferior** | 0.983 | 0.956–1.011 | 0.240 |
| **Right tubular-superior** | 1.016 | 0.985–1.049 | 0.306 |
| **Sex (Male)** | 5.317 | 2.080–14.236 | **<0.001** |
| **Age** | 1.026 | 0.938–1.123 | 0.580 |
| **Intracranial Volume** | 1.000 | 1.000–1.000 | 0.568 |

***Note:*** *Model AUC = 0.75*

**Table S2.** Exploratory false discovery rate (FDR) adjusted p-values for hypothalamic subregions in multivariable logistic regression models. Panel A reports results for the full model, whereas Panel B reports results after removal of influential observations based on Cook’s distance.

**Panel A.** Logistic regression results of the full model.

| **Variable** | **OR** | **CI** | **p-value** | **p_FDR** |
| --- | --- | --- | --- | --- |
| **Left anterior-inferior** | 1.051 | 1.000–1.106 | 0.051 | 0.171 |
| **Left anterior-superior** | 0.981 | 0.920–1.044 | 0.542 | 0.775 |
| **Left posterior** | 0.978 | 0.958–0.999 | 0.040 | 0.171 |
| **Left tubular-inferior** | 1.016 | 0.998–1.034 | 0.076 | 0.190 |
| **Left tubular-superior** | 0.990 | 0.971–1.010 | 0.325 | 0.650 |
| **Right anterior-inferior** | 1.001 | 0.949–1.057 | 0.956 | 0.956 |
| **Right anterior-superior** | 0.989 | 0.936–1.044 | 0.690 | 0.863 |
| **Right posterior** | 1.002 | 0.986–1.019 | 0.790 | 0.878 |
| **Right tubular-inferior** | 0.993 | 0.976–1.010 | 0.414 | 0.690 |
| **Right tubular-superior** | 1.024 | 1.005–1.044 | 0.013 | 0.129 |

**Panel B.** Logistic regression results of the Cook-cleaned model.

| **Variable** | **OR** | **CI** | **p-value** | **p_FDR** |
| --- | --- | --- | --- | --- |
| **Left anterior-inferior** | 1.073 | 1.016–1.133 | 0.012 | **0.039** |
| **Left anterior-superior** | 0.964 | 0.900–1.031 | 0.290 | 0.580 |
| **Left posterior** | 0.979 | 0.957–1.002 | 0.070 | 0.175 |
| **Left tubular-inferior** | 1.025 | 1.006–1.045 | 0.010 | **0.039** |
| **Left tubular-superior** | 0.993 | 0.972–1.015 | 0.535 | 0.764 |
| **Right anterior-inferior** | 1.009 | 0.952–1.070 | 0.773 | 0.773 |
| **Right anterior-superior** | 0.988 | 0.932–1.047 | 0.686 | 0.773 |
| **Right posterior** | 0.997 | 0.980–1.015 | 0.769 | 0.773 |
| **Right tubular-inferior** | 0.992 | 0.973–1.011 | 0.393 | 0.655 |
| **Right tubular-superior** | 1.034 | 1.014–1.056 | 0.001 | **0.012** |

**Table S3.** Variance inflation factors for the final Cook-cleaned logistic regression model.

| **Variable** | **VIF** |
| --- | --- |
| **Left anterior-inferior** | 1.524 |
| **Left anterior-superior** | 1.970 |
| **Left posterior** | 2.732 |
| **Left tubular-inferior** | 3.023 |
| **Left tubular-superior** | 2.694 |
| **Right anterior-inferior** | 2.015 |
| **Right anterior-superior** | 2.062 |
| **Right posterior** | 2.188 |
| **Right tubular-inferior** | 3.036 |
| **Right tubular-superior** | 2.156 |
| **Gender** | 2.207 |
| **Age** | 1.142 |
| **ICV** | 2.381 |


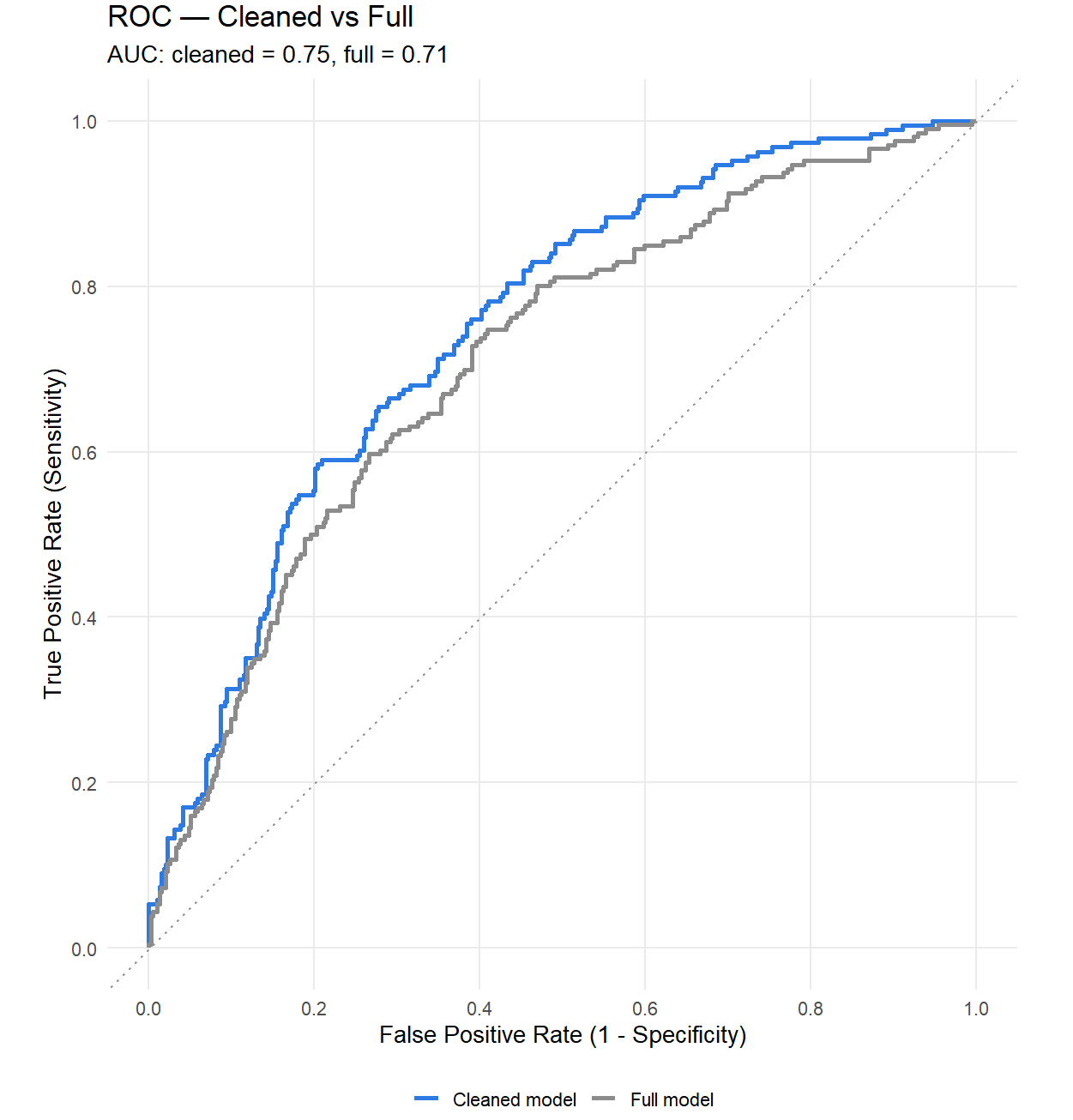


**Figure S1.** Receiver operating characteristic (ROC) curves for the multivariable logistic regression estimated on the full dataset (gray) versus the cleaned dataset after Cook’s distance removal (blue).


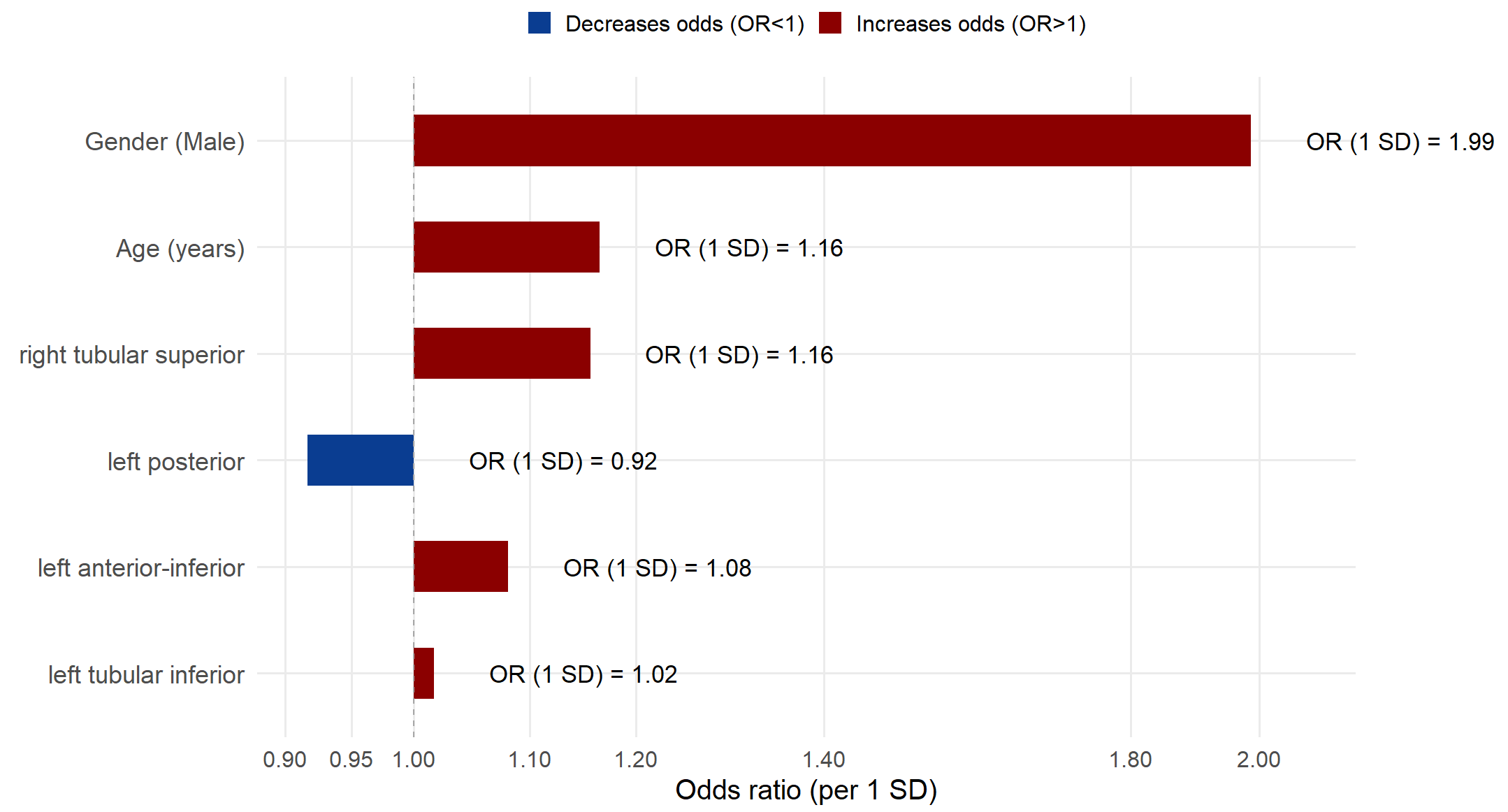


***Figure S2.*** *LASSO logistic regression for feature ranking. Bars report OR per 1 SD and are ordered by importance (|log OR|). Red bars indicate predictors where a 1-SD increase is associated with higher odds of being in the CC group (OR > 1). In contrast, blue bars indicate predictors where a 1-SD increase is associated with lower odds of being in the CC group (OR < 1).*
